# New products and seminar report

**DOI:** 10.1155/S1463924692000282

**Published:** 1992


Journal of Automatic Chemistry, Vol. 14, No. 4 (July–August 1992), pp. 151–156

### New products and seminar report

### LIMS

Hewlett-Packard's new laboratory information management software system, HP ChemLMS, operates under standard laboratory procedure language. Running on the UNIX-compatible HP-UX operating system under X-Windows, it is based on the industry-standard Oracle relational database management system. HP ChemLMS is intended for use in laboratories requiring a custom laboratory management system with true client/server capability that complies with regulatory standards.

HP ChemLMS is a highly flexible, language-based customization toolkit that can be tailored precisely to laboratory needs. Applications include research and development, quality control and assurance and general analysis in the chemical, pharmaceutical and consumer goods industries.

HP ChemLMS is the only laboratory management system that performs lab procedure revision and version control within the database and includes full audit-trail security. The audit-trail functions are integrated throughout the software and cover lab procedures as well as the data. With HP ChemLMS, lab management capability is extended to include the process by which data is collected. In addition, there is an option for automatic tracking and reporting of instrument maintenance and calibration records. These features help laboratories to meet regulatory requirements including Good Laboratory practice (GLP) and Good Manufacturing Practice (GMP).

HP ChemLMS incorporates an easy-to-use procedure specification language modelled on the language chemists use to write laboratory operating procedures. This allows HP ChemLMS to be customized by system integrators, HP application engineers or users to reflect exactly specific laboratory methods of working. As needs change, the software can easily be modified: chemists interact with the system just as they do with written laboratory procedures. Entering and viewing laboratory data is simple and flexible. Using the 'point-and-click' method with a mouse on multiple X-Windows viewing panels, users can view and enter data from various laboratory and corporate systems on a single terminal screen.

HP ChemLMS conforms to industry standards to provide customers with a stable laboratory management platform and easy access to a wide variety of other software packages from a variety of vendors.

*Enquiries to Verena Haller, Hewlett-Packard SA, 150 route du Nant-d'Avril, CH 1217 Meyrin (GE) 2, Switzerland.*

### Elemental Analysers

Carlo Erba Instruments, part of the Organic Division of Fisons Instruments, has recently introduced a new generation of organic elemental analysers: the EA 1108 and the NA 1500 Series 2. Both systems analyse solid and liquid samples in organic and inorganic materials. They can simultaneously determine concentrations from 100 ppm to 100%. The systems do not require special operator intervention after sample weighing. Autosamplers are available (up to 196 unattended analyses) for solids and liquids, direct liquid injection, and gases.

The operating principle of flash combustion followed by gas chromatographic separation and detection allows the instruments to be directly coupled to other detectors, such as an Electron Capture Detector, for trace sulphur analysis, or a mass spectrometer for isotopic ratios determination.

The NA 1500 Series 2 gives fast, accurate simultaneous determination of nitrogen, carbon and sulphur. The system is available in three analytical configurations – nitrogen (protein), nitrogen/carbon, or nitrogen/carbon/sulphur.

The EA 1108 determines the concentration of four elements: carbon, hydrogen, nitrogen and sulphur simultaneously and can be switched to direct oxygen measurement. With a flexible design, CHN, CHNS, CHN-O, and CHNS-O versions are available to meet the needs of petrochemical, pharmaceutical, and organic chemical applications.

*More information from Fisons Instruments, Sussex Manor Business Park, Gatwick Road, Crawley, Sussex RH10 2QQ, UK.*

### Liquid-solid extractor

A liquid-solid analytical extractor that uses standard Soxhlet glassware

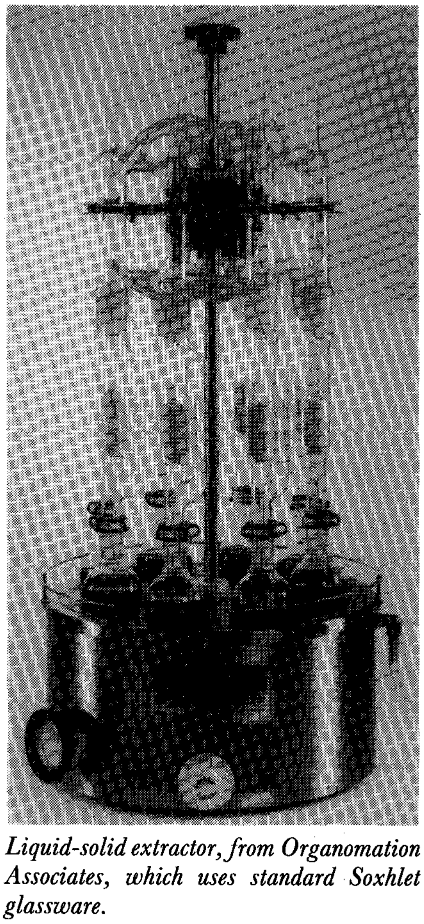

*Liquid-solid extractor, from Organomation Associates, which uses standard Soxhlet glassware.*

and accommodates either Friedrichs and Allihn condensers has been introduced by Organomation Associates, Inc. The ROT-X-TRACT Model 130 liquid-solid extractor handles up to eight standard 125, 250, or 500 ml Soxhlet glassware units and either Friedrichs or Allihn condensers in a circular arrangement. Featuring a stainless-steel bath and a condenser water flowmeter, the unit is equipped with a thermostat that can hold bath water temperature to within ±2 °C.

Providing complete access to all glassware from the front, the glassware units on Organomation's ROT-X-TRACT Model 130 are connected to a circular parallel water manifold mounted on a centre tube. Requiring only 356 mm bench space, it has an overall height of 813 mm. Options include an intrinsically safe steam bath or hot plates for boiling high temperature solvents.

Organomation's ROT-X-TRACT Model 130 is priced according to user glassware and condenser requirements.

*More information from Organomation Associates, Inc., Andrew McNiven, Marketing, 266 River Road West, Berlin, MA 01503-1699, USA. Tel.: 508 838 7300; fax: 508 838 2786.*

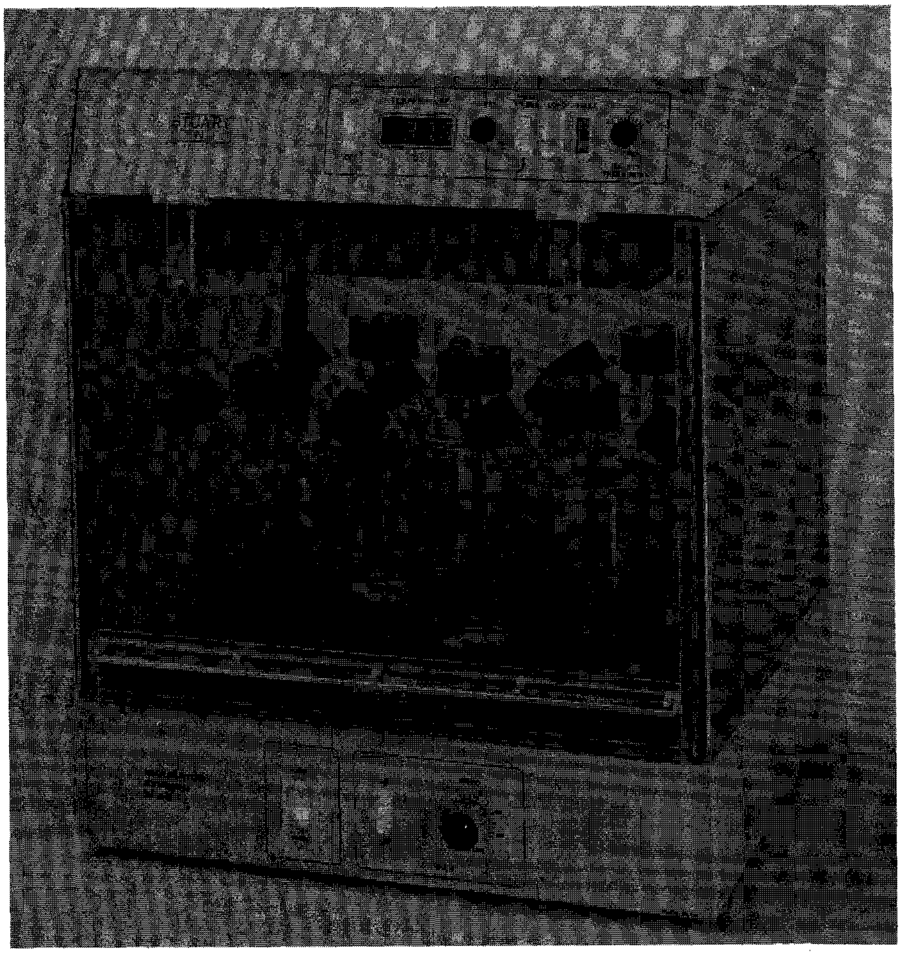

*The new SI 40 Magnetic Stirrer Incubator is a bench-top incubator which incorporates a in-built magnetic stirrer. The SI 40's six-place biological magnetic stirrer will accommodate vessels up to 280 mm in height, including culture flasks, and stir them at any set speed between 10 and 100 rpm. An important feature of the stirrer is electronic feedback speed control. This ensures a soft start to stirring, and also accurately maintains the set speed, even with changes in liquid viscosity. Details from Stuart Scientific, Holmethorpe Avenue, Holmethorpe Industrial Estate, Redhill, Surrey RH1 2NB, UK.*

### Seminar report

*Automatic Data Acquisition. Held on 26 and 27 March 1992 in Nairobi, Kenya*

A two-day seminar on Automatic Data Acquisition was held in Nairobi, Kenya, organized by a local electronic engineering company. The seminar was fully sponsored by United States Agency for International development (US-AID); and number of participants was limited to 25.

The participants were mainly from national and international biomedical research institutions based in Kenya; a major multinational engaged in the agro-industry also sent delegates from, for example, the factory maintenance, production support and quality-control divisions. Professionals from a variety of disciplines attended, for example, the group included a chemist, biometrician, soil scientist, and medicinal chemist.

On the first day of the seminar, the opening subject was on concepts and applications of data acquisition. A presentation on how to interface instruments to computers followed. The analysis and management of data was then discussed and the day ended with discussions reviewing the day's presentations and an introduction on the analysis of a user's requirement in specifying a data acquisition system.

The second day started with two presentations – on design methodologies and implementation strategies. The afternoon was devoted to discussions on problems or potential areas of applications as envisaged by the participants. The seminar was concluded with a review of the two days and the award of certificates to participants.

Demonstrations available included a blood analyser/sampler and various commerical laboratory automation software.

The commonality of data acquisitions systems on a variety of applications became apparent during discussions. The question of cost-effectiveness, especially in the Third World where hardware and software, if available, can be prohibitingly expensive, was always in the mind of the participants throughout the discussions. The presenters mentioned that 'home-made' software may be affordable, considering the lower local manpower costs. Also highlighted was the fact that some interface equipment could be designed and produced locally, with the advantages of reduced costs a major attraction, but also the close proximity of the designer to the user, enabling a better understanding and fulfillment of the design and user requirements. Here the 'openness' of PC-based systems was emphasised.

The feedback from participants suggested that more such seminars would be helpful, especially in raising the awareness of not just the solutions, but of local personnel to design and implement the solutions.

*Further details are available from Dr J. S. O. Odonde, PO Box 54, 4542 79 Hoek, The Netherlands.*

### Water information on CD-ROM

Microinfo has announced two further database services on CD-ROM.

Aqualine is produced by Britain's Water Research Centre and is published on CD-ROM by Compact Cambridge, under licence from Pergamon Press. Waterlit, also published on CD-ROM by Compact Cambridge, is a product of the South African Water Information Centre. The Water Reseach Centre, which compiles and indexes the Aqualine database, provides technical leadership in support of water policies and undertakings around the world. Information contained in Aqualine corresponds in part to *Aqualine Abstracts.*

Aqualine provides more than 30 years of references to the world's literature in water-related studies. Encompassing over 138 000 citations and abstracts, topics covered include water resources and supplies; water quality; chemical analysis and monitoring of water and wastes; water and waste-water treatment; underground services and water use; sewage; industrial effluents; effects of pollution; instrumentation control and computing and appropriate technology.

Personnel at the South African Water Information Centre scan 500 journals from around the world to select specific information for Waterlit. Approximately 12 000 records are added to the database each year. Waterlit adds a valuable dimension to studies of aquatic resources that will prove indispensable for anyone involved in the science and technology of water, waste-water and sanitation.

Waterlit contains over 185 000 citations and abstracts to the world's literature on water, waste-water and sanitation. Covering 1975 to the present, the database provides information on subjects ranging from distribution to drought evaluation; from leak detection to legal issues.

Aqualine offers 18 searchable fields. These include author, title, journal name, subject terms, type of publication and major subject area. Two indexes allow multiple fields to be searched simultaneously. Waterlit offers 22 searchable fields – these include author, title, source, publication year, conference, subject heading, classification and descriptor. Two indexes allow simultaneous searching of multiple fields.

*For further information including details of current subscription rates please contact CD-ROM Division, Microinfo Ltd, PO Box 3, Omega Park, Alton, Hampshire GU34 2PE, UK.*

### WaveTrak 2.1 software

WaveTrak 2.1 software for the Macintosh had been designed for scientific/technical applications requiring the acquisition, storage and manipulation of digitized traces. Using previously available tools, locating a specific waveform after the event could take as much time as the original experiment. WaveTrak solves that problem by storing each separate digitized trace on a separate card of a HyperCard stack. In addition to the data, the card can contain a time stamp, hardware parameters (system gain, temperature, sampling rate etc.) and a user-supplied comment to annotate a specific peak or region of the trace. These annotations can be searched, allowing virtually instant retrieval of a specific piece of information. Each card can contain up to 30 000 data points. The number of cards in a stack is limited only by the available disk storage space. Waves are displayed at screen resolution, and a unique zoom feature allows full 30 000 point resolution for selected regions of interest.

Once data is recorded into a stack it can be duplicated and distributed for viewing or analysis using only the basic HyperCard 2.0 program, making the program ideal for teaching or student use. Traces may be exported in TEXT, PICT or IGOR format.

In addition to coveniently eliminating the need to clutter up a hard disk with hundreds of separate files and folders, WaveTrak provides a library of buttons that allow the user to customize mathematical or display routines for a particular application. Standard buttons include routines for FFT, DV/DT, pulse generation, and many more. WaveTrak supports use of the DACS on the MacADIOS board, as well as the digital I/O lines, for easy construction of input/output protocols. HyperCard-conversant users can also create their own buttons, menus and cards using HyperTalk scripts. More experienced users can create highly specialized custom functions by writing their own XCMDs or XFNDs in Pascal, C, or assembly language.

To acquire data, WaveTrak requires a Macintosh II series CPU with a hard disk and at least 2 MB or RAM, math coprocessor, and the GW Instruments (Somerville, MA) MacADIOS II NuBus card. Display and data manipulation requires only a Macintosh computer wiht HyperCard 2.0.

*WaveTrak is distributed by MacScience Solutions, PO Box 205, Hopedale MA 01747, USA. Tel.: 508 478 6887.*

### Thermogravimetry

In thermogravimetric analyses, mass changes of a sample are measured as a function of temperature or time. Exactly weighed samples are subjected in a furnace to specified temperature/time programs under strict

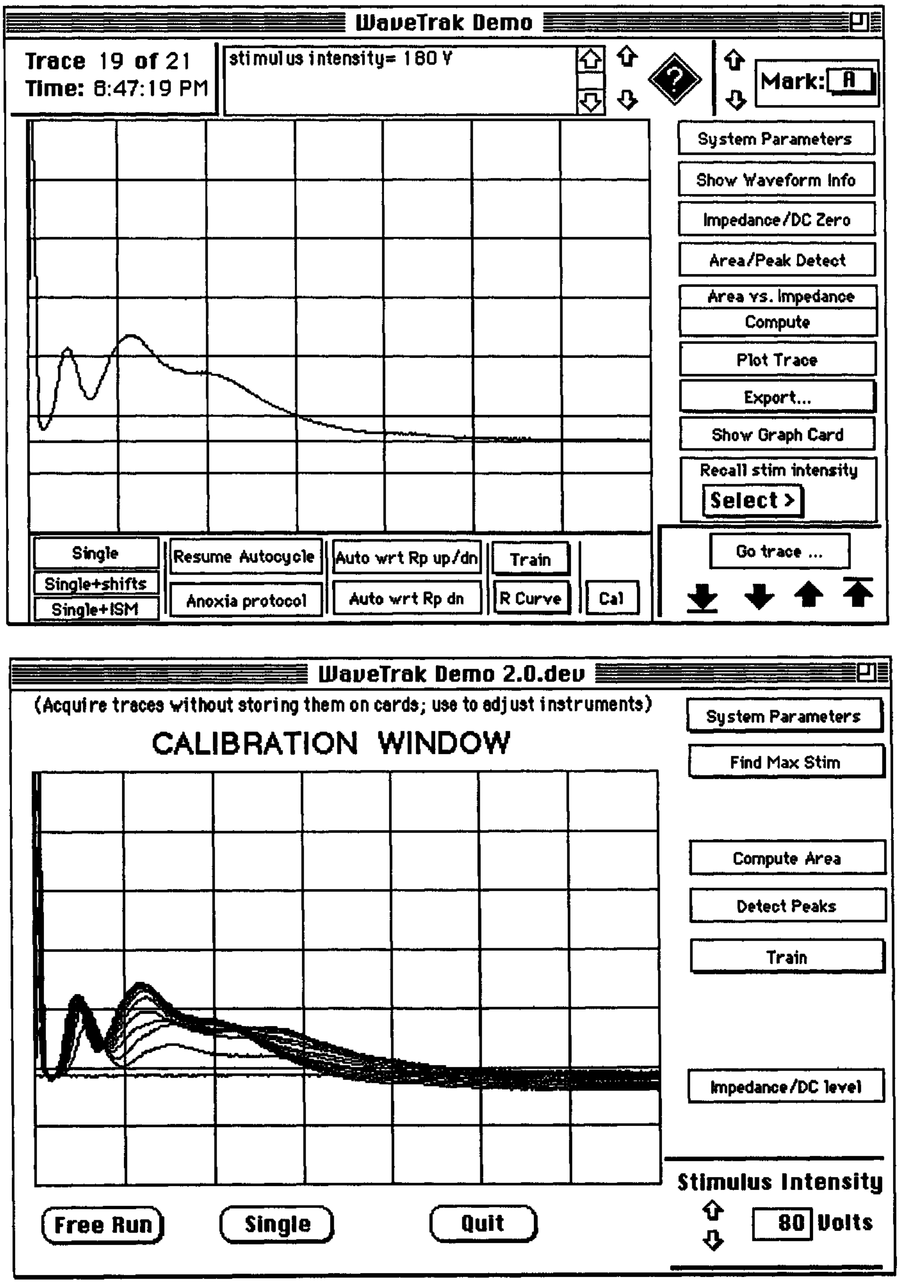

*WaveTrak 2.1 software for the Macintosh is intended for acquiring large numbers of digital waveforms and storing them for future reference.*

temperature control. Mass changes during the analysis are continuously recorded by a microbalance. Thermogravimetic analyses represent a simple way to obtain information on the nature, composition and thermal stability of substances and for investigating reaction kinetics.

The microbalance installed in the new MT5-TG/TG50 Measuring Cell mirrors the ergonomic design of the Mettler analytical balances of the AT series. This is the first time that a continuous weighing range of 5000 mg has been achieved. The balance is self-calibrating, thereby assuring dependability over the entire weighing range. Rugged construction and overload protection make the operation straightforward. Data interfaces built in as standard allow the weighing cell to communicate with other devices in a LIMS. A multifunctional LCD offers comprehensive information on all aspects of the configuration and operation. The time-tested DeltaTrac provides information in analogue form on the available weighing range. During periods of inactivity, the microbalance can be employed without restrictions as a stand alone microbalance, for example for weighings of DSC samples.

*Details from Mettler-Toledo AG, CH 8606 Greifensee, Switzerland. Tel.: 01 944 221.*

### Graphite furnace

GBC Scientific Equipment's SYS3000 graphite furnace system is a fully automatic, multi-element instrument. The SYS3000 accomodates an eight-lamp turret and an automatic samples changer and is able to determine up to 12 elements in a batch of samples without operator intervention. Samples which exceed the dynamic range of the calibration curve are automatically diluted until they can be accurately analysed.

The system is controlled by an IBM AT Compatible or Personal System/2 Model 50 Computer with high resolution, colour graphics and hard disk storage of operating parameters and results. The hard disk also enables storage of the signal graphics traces for all samples in a multi-element run. The operator can then view the signal, background or temperature profile for any sample and zoom in if necessary.

*Details from Orry Dugdale, GBC Scientific Equipment (UK) Ltd, 13 Frederick Sanger Road, The Surrey Research Park, Guildford, Surrey GU2 5YD, UK. Tel.: 0483 304988; fax: 0483 303071.*

### FIA system

The Lambda FIA System from Perkin-Elmer consists of the FIAS 200 high-performance flow-injection system for atomic spectroscopy with two separately programmable pumps, the AS 90 autosampler with separate trays for up to 152 samples and the Lambda 2 UV/VIS scanning spectrometer. Included in the system is a detailed cookbook, outlining analytical protocols for the determination of important anions and other analytical parameters. The Lambda 2 serves as the FIA detector and can also be used as a conventional UV/Vis spectrometer. It has a wavelength range from 190 to 1100 nm and is fully PC-controlled, using the specialized PEFIA software package with 26 preprogrammed methods for flow injection analysis with the Lambda FIA System.

The system should be of particular interest to scientists involved in environmental, drinking- and waste-water, pharmaceutical and food analyses.

*For further information, contact Perkin-Elmer Limited, Post Office Lane, Beaconsfield, Buckinghamshire HP9 1QA, UK. Tel.: 0494 676161; fax: 0494 679061.*

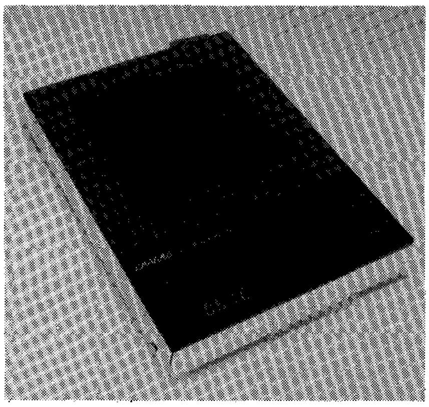

*The CAMAG TLC Plate Heater III is designed for heating a TLC plate to a given temperature after a derivatization reagent has been applied. The temperature is uniformly maintained over the entire area. The TLC Plate Heater III has a CERAN ceramic hotplate and is easily cleaned. The 200 × 200 mm heating area has a grid to facilitate correct positioning of the TLC plate; the temperature range is 25–200 °C. Details from CAMAG, Sonnenmattstrasse 11, CH 4132 Muttenz, Switzerland.*

### HPLC training software

*Method Development in High Performance Liquid Chromatography* is a comprehensive, animated training programme designed to operate with Microsoft Windows. The programme deals with the selection of columns and detectors, sample preparation, eluent survey, separation optimisation, qualitative and quantitative analysis and preparative methods. The interactive programme has action sequences in full colour and includes quizzes for on-screen self testing. 'Hot words', accessed with the mouse, provide on-screen definitions of important chromatographic terms.

*Details from Autoscribe Ltd, PO Box 5, Riseley, Reading RG7 1YL, UK. Tel.: 0734 883125; fax: 0734 885604.*

### Scientific software

Europa Scientific Software Corporation (es$^2$c) serves the specialized software needs of the scientific research community in North America and Europe. es$^2$c has a new catologue, which offers scientists a unique selection of over 200 specialized software packages for the IBM PC (or compatibles) and the Macintosh from more than 60 different scientific software developers. The products featured in the es$^2$c catalogue include specialized drawing tools for chemists and chemical engineers, presentation graphics for slides and posters, sophisticated molecular modelling, mathematical modelling, statistics and data analysis, scientific graphing and plotting, bibliography management, and translation software. The specialized scientific programs are augmented by commonly used programs for the PC and Macintosh, such as Lotus 1-2-3, Cricket Graph, Microsoft Word and Excel etc.

*More information from Europa at PO Box 1114, Hollis, NH 03049, USA. Tel.: and fax: 60 465 2811.*

### Super fluid extractor

Hewlett-Packard has announced an enhancement to its succesful supercritical fluid extractor (SFE), the HP

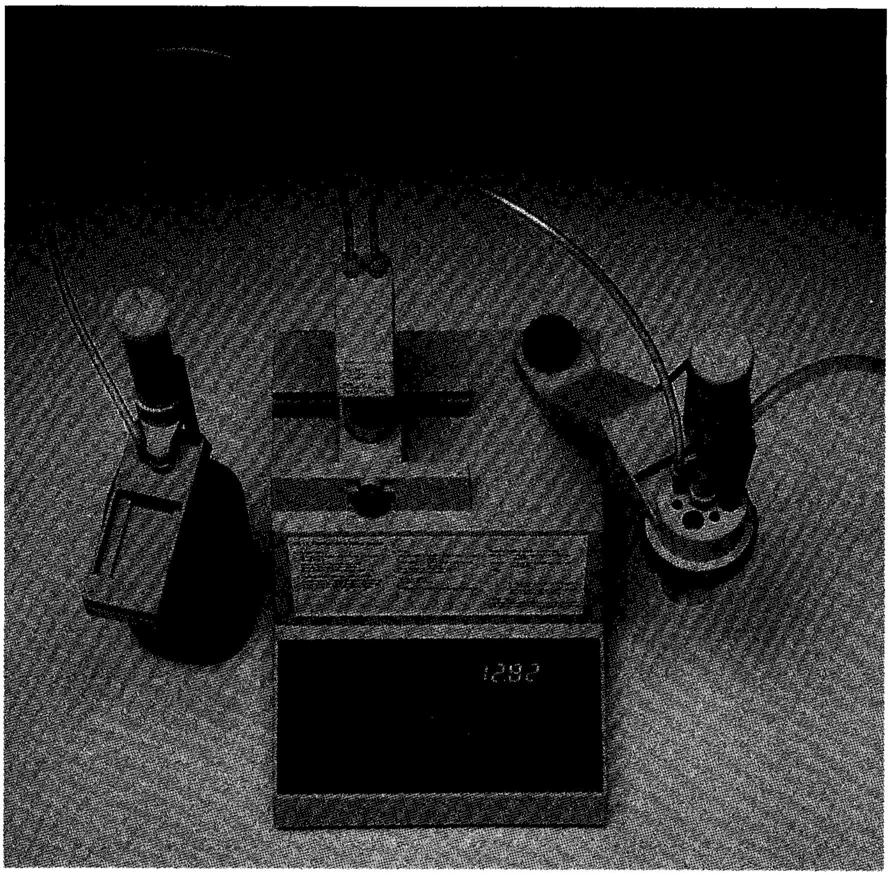

*The Mettler DL12 is intended for routine analyses with high demands on accuracy; the DL12 is especially suitable for pH titrations and is moderately priced. Operation of the DL12 is simple and user friendly. The sample size is automatically taken into account in the calculation of the results. A new feature is the possibility to obtain the result with inputted constants or with a preprogrammed code (19 variations) in the desired mass unit. The titration routine is started at a keystroke. The simple method concept assures a high degree of reproducibility of the results. Details from Mettler-Toledo AG, CH 8606 Greifensee, Switzerland.*

7680A, which was introduced to the analytical market two years ago.

The HP 7680T, designed to simplify the preparation of solid and semi-solid samples in the chemical, pharmaceutical, environmental, food and flavour industries, provides totally automated sample preparation from sample loading through to sample delivery. A series of eight samples can be run automatically. The system's single controlling personal computer can run two SFE units, allowing up to 16 samples to be extracted automatically. Each sample can be run under different conditions for automated method development; or under the same method for high throughput.

The HP 7680T SFE is a graphics-driven device that operates on a personal computer-based PH ChemStation. The HP ChemStation also serves as an all-purpose, multi-tasking laboratory computer for word processing, spreadsheets, data management and chromatography data handling.

*Enquiries to Verena Haller, Hewlett-Packard SA, 150 Route de Nant-d'Avril, CH-1217 Meyrin (GE) 2, Switzerland. Tel.: 41 22 780 8227.*

### Deconvolution application

Galactic has introduced an advanced curve deconvolution application based on the Levenburg-Marquardt non-linear least squares minimization algorithm. The application runs under Galactic's Windows-based software, GRAMS/386, and is designed to deconvolve and separate overlapped peaks. GRAMS Curvefit can fit Gaussian, Lorentzian, Mixed Gaussian plus Lorentzian, Pearson VII, Voight, and Log Normal peaks, and it will fit baselines from simple offset to cubic polynomials. Fitted peak types can be mixed, positive or negative, and the number of fitted peaks is virtually unlimited. The processing power of GRAMS' optimized 32 bit application instructions gives GRAMS curvefit extraordinary speed. The fit interations are complete with unmatched efficiency, and can be performed continuously or one step at a time to monitor the progress of the fit.

Initial peak parameters can be set either manually or automatically. Once the fit is complete, data can be viewed singly, with the base-line, with the residual, and it can be overlaid. Full statistical information including standard errors of the parameters, standard errors of the fit, and peak areas are provided. This information and actual fitted data can be easily output to hardcopy or pasted into other applications.

*For additional information contact Kelly W. McIntire, Galactic Industries Corporation, 395 Main Street, Salem, NH 03079, USA. Tel.: 603 898 7600; fax: 603 898 6228.*

### Syringeless filters

Whatman Scientific's syringeless filters have been designed to simplify the preparation of small volume samples and can be used in automated techniques. Three types of device are available, each with a choice of filter media.

Autovial contains a 25 mm diameter filter unit in a specially designed barrel housing. Samples are placed in the graduated barrel (12 ml capacity) and filtered directly by depressing the self-sealing plunger.

Uniprep and Cliniprep operate by means of a hollow plunger which incorporates the filtration medium in its base. Samples are placed in the device's outer tube and filtered by means of inserting the hollow plunger. This action creates a vial of sample which can be removed, capped and used directly in further analytical procedures.

Uniprep is available with a choice of media for general laboratory applications: PVDF, PTFE, Nylon 66 and glass microfibre in 0·2 and 0·45 µm pore sizes.

The Cliniprep range offers a choice of optimized combinations of filter media. CliniPrep-BAC is for stool samples, CliniPrep-GEN for general clinical samples, CliniPrep-STD for mucous and CliniPrep-REN is for fine filtration of urine or serum specimens, especially useful for testing for drugs of abuse.

*For further information contact Whatman Scientific Ltd, Whatman House, St. Leonard's Road, 20/20 Maidstone, Kent ME16 0LS, UK. Tel.: 0622 676670; fax: 0622 677011.*

